# A comparison of the efficacy of antiangiogenic agents combined with chemotherapy for the treatment of non-small cell lung cancer: a network meta-analysis

**DOI:** 10.1186/s12935-020-01639-4

**Published:** 2020-11-10

**Authors:** Yimin Li, Yonglin Yi, Anqi Lin, Peng Luo, Jian Zhang

**Affiliations:** Department of Oncology, Zhujiang Hospital, Southern Medical University, 253 Industrial Avenue, Guangzhou, 510282 Guangdong China

**Keywords:** Antiangiogenic agents, Bevacizumab, Endostar, Non-small cell lung cancer, Network meta-analysis

## Abstract

**Objection:**

To explore the effects of combinations of antiangiogenic agents and chemotherapy agents on non-small cell lung cancer (NSCLC) patients and indirectly compare the therapeutic effect of Endostar combined with chemotherapy and bevacizumab combined with chemotherapy on NSCLC.

**Methods:**

We searched 3 electronic databases: PubMed, Web of Science and the Cochrane Library. The ORRs, HRs and 95% confidence intervals of OS and PFS were used to compare the efficacy of Endostar combined with chemotherapy and bevacizumab combined with chemotherapy. We use the Bayesian network meta-analysis method to make indirect comparisons and obtain rank probabilities; in addition, we used single-arm meta-analysis to synthesize the existing data.

**Results:**

A total of 29 studies were included in the analysis. Among them, we included a total of 14 interventions. A total of 12,862 patients participated in this analysis. The single-arm meta-analysis showed that the pooled ORR and 95% CI were 0.35 (0.31, 0.39), the pooled HR of OS and 95% CI were 0.89 (0.81, 0.98), and the pooled HR of PFS and 95% CI were 0.67 (0.56, 0.81). According to the results of network meta-analysis, there were no significant differences between the 5 kinds of bevacizumab combined with chemotherapy regimens and the 4 kinds of Endostar combined with chemotherapy regimens for improving ORR and prolonging OS and PFS. The rank probabilities suggested that in terms of ORR, Pla + Pem + Bev was the first-ranked intervention (0.288). Pla + Pem + Endo was the first-ranked intervention for prolonging OS (0.423) and Pla + Gem + Endo was the first-ranked intervention for prolonging PFS (0.302).

**Conclusion:**

Antiangiogenic agents combined with platinum-containing dual drugs can provide benefits to NSCLC patients. In addition, bevacizumab combined with chemotherapy regimens has better theraputic effect on ORR while Endostar combined with chemotherapy may have better effects on OS and PFS for the treatment of NSCLC patients.

## Background

Lung cancer has the highest incidence rate of cancer in developed countries and is the leading cause of cancer-related death in the United States [1]. Non-small cell lung cancer (NSCLC) is the main pathological type of lung cancer, accounting for 85% of all types, and includes squamous cell carcinoma, adenocarcinoma, large cell carcinoma and adenosquamous carcinoma. Angiogenesis, which was proposed by Folkman in 1971 [2] and listed as one of the hallmarks of tumors by Hanahan [3], plays an important role in the occurrence and development of tumors. Angiogenesis is related to the proliferation, invasion and metastasis of tumors [4]. Many molecules, such as vascular endothelial growth factor (VEGF), platelet-derived growth factor (PDGF), transforming growth factor-β (TGF-β), fibroblast growth factor (FGF) and other important molecules, participate in the process of angiogenesis [5]. Currently, with various antiangiogenic agents being approved for cancer treatment, antiangiogenic therapy for NSCLC has attracted increasing attention.

Bevacizumab is a humanized monoclonal antibody with high affinity to VEGF [6]. The VEGF family includes VEGF-A, VEGF-B, VEGF-C, VEGF-D and placental growth factor, which have been suggested to be overexpressed in NSCLC, providing necessary conditions for angiogenesis [7]. Bevacizumab has a high affinity and specificity for VEGF, and therefore, it can inhibit the growth of NSCLC [6], which provides a premise for it to become an agent for the treatment of NSCLC. The results of E4599, which is a randomized, controlled, multicenter clinical trial, show that bevacizumab combined with carboplatin and paclitaxel can significantly improve the response rate and prolong progression-free survival (PFS) and overall survival (OS) compared with carboplatin and paclitaxel alone for patients with unresectable and advanced non-squamous cell carcinoma who were chemotherapy naive [8]. Based on this finding, in 2005, the Food and Drug Administration approved bevacizumab combined with carboplatin and paclitaxel for the first-line treatment of advanced non-squamous cell carcinoma [9]. However, at present, only bevacizumab combined with cisplatin and paclitaxel has been approved for NSCLC treatment, and the differences in outcomes of bevacizumab combined with different chemotherapies have not been clearly determined.

Endostatin is a kind of natural protein that was first isolated and extracted by Judah Folkman in mouse tumor strains and showed the strong antiangiogenic and tumor growth inhibition effects [10]. Endostatin has a wide antitumor spectrum [11]. The specific antiangiogenic mechanism has not been fully elucidated at present. The main mechanism of the antitumor effect is that endostatin acts on the VEGF receptor KDR/Flk-1 and inhibits the signal transduction of VEGF, which therefore inhibits angiogenesis [12]. Endostar is a recombinant human endostatin with 9 added amino acids (MGGSHHHHH) [13] to maintain stability and a long half-life. A randomized, double-blind, controlled, multicenter phase III clinical trial from China showed that the combination of Endostar with vinorelbine and cisplatin can significantly improve the response rate, median time to progression (TTP), and quality of life of patients with advanced NSCLC compared with vinorelbine and cisplatin alone. There were no significant differences in adverse events, but its cardiotoxicity needs further attention [14]. Endostar was approved by the China Food and Drug Administration in 2005 for the treatment of NSCLC [15]. However, at present, only one phase 3 clinical trial has shown that the efficacy of Endostar combined chemotherapy, and the advantages of Endostar combined with other chemotherapeutics need more rigorous clinical trials for confirmation.

Currently, it is still not clear that antiangiogenic drugs combined with which chemotherapy regime can provide the most benefit to NSCLC patients. Besides, although the results from phase 3 clinical trials showed that NSCLC patients can benefit from bevacizumab combined with chemotherapy and Endostar combined with chemotherapy for first-line treatment, there is a lack of head-to-head clinical trials of the two schemes. Therefore, we used the Bayesian network meta-analysis method to indirectly compare the efficacy of bevacizumab combined with chemotherapy and Endostar combined with chemotherapy in the treatment of NSCLC. In addition, we used single-arm meta-analysis to synthesize published clinical trial results to comprehensively evaluate the therapeutic effect of bevacizumab combined with chemotherapy and Endostar combined with chemotherapy on NSCLC patients.

## Materials and methods

### Search strategy and eligibility criteria

On May 1, 2020, we used the following key words to retrieve literature from the PubMed, Web of Science and the Cochrane Library databases: "Anti-VEGF Humanized Monoclonal Antibody", "anti VEGF monoclonal antibody", "rhuMAb-VEGF"; "Endostar", "recombinant human endostatin", "Rh endostatin", "YH-16"; "Avastin", "bevacizumab"; "Cyramza", "Ramucirumab", "Brigatinib", "Alunbrig", "Cabozantinib", "Cabometyx", "Cometriq"; "non small cell lung cancer", "Lung cancer" and "NSCLC". All MeSH terms and entry terms were used to achieve a comprehensive search. In this search, there were no restrictions on the language or publication date.

Inclusion and exclusion criteria were established for the purpose of our analysis. The inclusion criteria were as follows: the study type was a clinical trial or prospective study; the study population was NSCLC patients who had not received chemotherapy or NSCLC patients who had completed previous chemotherapy for more than or equal to 4 weeks; the interventions were bevacizumab combined with chemotherapy, Endostar combined with chemotherapy or chemotherapy alone; the study design involved studies with at least two arms and included at least the above two interventions or included the same intervention in the same study but used different chemotherapy agents; and studies that provided at least one of the following outcome measures: objective response rate (ORR), overall survival (OS), and progression free survival (PFS), studies that provided the event rate of adverse events after treatment were also included. The exclusion criteria included articles that could not be provided in full-text and non-English articles; and the following article types were also excluded: review articles, case reports, meeting abstracts, meta-analyses, cell animal experiments, etc. Three reviewers (YL, YY and AL) independently performed preliminary screening according to the titles and abstracts and then confirmed whether to include the studies by reading the full texts according to the inclusion and exclusion criteria. Any disputes regarding the included studies were resolved through discussion with the fourth reviewer (PL).

### Data extraction

Three reviewers (YL, YY and AL) used a previously designed data extraction table to extract data independently. The extracted data included the general data of the study (such as the first author, study year, proportion of males, treatment plan, performance status, proportion of squamous cell carcinoma), the event rate of adverse events, the ORRs, hazard ratios (HRs) and 95% confidence intervals (CIs) of OS and PFS. If the HR and 95% CI were not provided in the study, we used Tierney's method to estimate them [16]. Any disputes or inconsistencies were discussed with the fourth reviewer (PL).

### Quality assessment

Three reviewers (YL, YY and AL) independently evaluated the quality of the research methods of the included studies. The Cochrane Collaboration's risk of bias (ROB) tool was used to evaluate the quality of randomized controlled trials [17], and the Newcastle Ottawa Scale (NOS) was used to evaluate the quality of nonrandomized controlled trials and prospective studies [18]. For randomized controlled trials, if there were more than four "low risk" domains based on the ROB tool, the study was considered to have "high quality"; if there were two or more "low risk" domains, the study was considered to have "moderate quality"; and finally, if there were less than two "low risk" domains or more than one "high risk" domain, the study was considered to have "low quality". For nonrandomized controlled trials, if the total NOS score was 7–9, the study was considered to have "high quality"; if the total NOS score was less than 4, the study was considered to have "low quality"; and if the total NOS score was 4–6, the study was considered to have "moderate quality". Similarly, any disputes and inconsistencies were discussed with the fourth reviewer (PL).

### Statistical analysis

R 3.5.1 software (The R Foundation, Vienna, Austria) was used for Bayesian meta-analysis. For the binary variable (ORR) and time-to-event data (HRs of OS and PFS), we used the "gemtc" package and "JAGS" package of R software, using the Markov chain Monte Carlo method [19] to simulate four different chains, with 40,000 iterations, 160,000 burn-ins and a thinning interval of 10. The Bayesian network meta-analysis was carried out so that each intervention could be compared indirectly, and the rank probabilities of various interventions can be obtained. For the comparison of ORR, the results are shown as odds ratios (ORs) and 95% credible intervals (CrIs); for the comparison of OS and PFS, the results are shown as HRs and 95% CrIs, and the results of all CrIs were bilateral. In addition, to ensure the credibility of the results, we used the "gemtc" package and "JAGS" package in R 3.5.1 software to analyze the ORR and HR of OS and PFS by nodesplit analysis to explore the consistency between direct comparisons and indirect comparisons. P > 0.05 indicates that the difference between the direct comparison and the indirect comparison was not statistically significant. P < 0.05 indicates that the difference between the direct comparison and the indirect comparison was statistically significant. Heterogeneity analysis was used to compare the degree of heterogeneity in the research results. When the P value was more than 0.1 and I^2^ was less than 50%, the heterogeneity was not statistically significant. When the P value was less than 0.1 and I^2^ was more than 50%, the heterogeneity was statistically significant.

Review Manager 5.3.4 software (Cochrane Library, Oxford, UK) and STATA 14.0 software (Stata Corp., College Station, TX) were used for single-arm meta-analysis. The ORR, OS, PFS, HRs of OS and PFS and the event rate of adverse events were used to obtain pooled results. The statistical model was selected according to the degree of heterogeneity. When the P value was more than 0.1 and I^2^ was less than 50%, no statistically significant heterogeneity was indicated, so a fixed effects model was used. When the P value was less than 0.1 and I^2^ was more than 50%, statistically significant heterogeneity was indicated, so a random effect models was used. At the same time, we used RevMan to evaluate publication bias intuitively through funnel plots and STATA to evaluate publication bias quantitatively through Begg’s and Egger's tests. In addition, STATA was used to generate a network plot to describe the number of patients included in the study and the number of head-to-head comparisons.

## Results

### Characteristics of the included studies

**Fig. 1:**
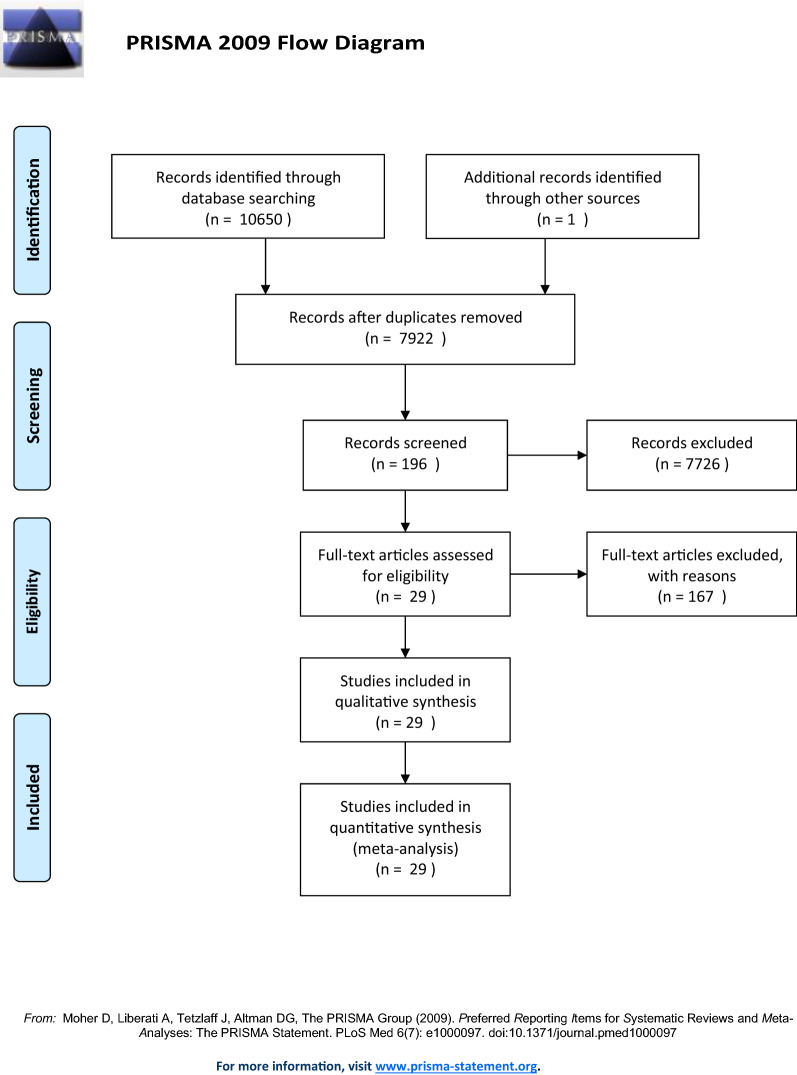
The flow diagram of the study selection process for the network meta‐analysis

According to the search strategy, we retrieved 10,650 articles in PubMed, Web of Science and the Cochrane Library. Sun Yan et al. provided inadequate information [47] for our network meta-analysis, and therefore, we e-mailed the corresponding authors for further information. Finally, the author provided us with more detailed results of a phase 4 clinical trial of Endostar as additional sources for retrieval. Finally, a total of 29 articles were included in our study, including 4 nonrandomized controlled trials and 25 randomized controlled trials. The screening process of the articles is shown as a flow diagram in Fig. 1. The general characteristics of the patients in each study are shown in Table 1. The details of quality assessment are shown in Additional file 1: Figure S1.

**Table 1:** General characteristics and quality assessment of the included studies

Study ID	Treatment	Sample size	Median age	Male (%)	Stage IV (%)	SCC (%)	PS = 2	Quality assesment
Boutsikou [20]	Docetaxel (100 mg/m^2^) + Carboplatin (5 mg/mL*min) + Bevacizumab (7.5 mg/kg)	56	63	80.4	73.2	0	0	Moderate
	Docetaxel (100 mg/m^2^) + Carboplatin (5 mg/ mL*min)	61	65	85.2	83.6	0	0	
Fukuda [21]	Bevacizumab (15 mg/kg) + pemetrexed (500 mg/m^2^)	20	78.5	55	75	0	0	Moderate
	pemetrexed (500 mg/m^2^)	20	77.5	60	75	0	0	
Galetta [22]	Paclitaxel (200 mg/m^2^) + Carboplatin (6 mg/mL*min) + Bevacizumab (15 mg/kg)	58	62	77.6	93.1	0	0	High
	Pemetrexed (500 mg/m^2^) + Cisplatin (75 mg/m^2^)	60	60	70	95	0	0	
Gronberg [23]	Gemcitabine (1,000 mg/m^2^) + Carboplatin (6 mg/ mL*min)	217	66	59.0	71.9	23.0	22.6	High
	Pemetrexed (500 mg/m^2^) + Carboplatin (6 mg/ mL*min)	219	64	56.2	71.2	26.0	21.5	
Han [24]	Paclitaxel (175 mg/m^2^ on day 1) + Carboplatin (5 mg/ mL*min on day 1) + Endostar (7.5 mg/m^2^/d on days 8 and 21)	61	57	80.3	70.5	37.7	6.6	High
	Paclitaxel (175 mg/m^2^ on day 1) + Carboplatin (5 mg/ mL*min on day 1) + Endostar (7.5 mg/m^2^/d on days 8 and 21)	61	58	62.3	59	23	3.3	
Johnson [25]	Paclitaxel (200 mg/m^2^) + Carboplatin (6 mg/mL*min) + Bevacizumab (15 mg/kg)	35	57	45.7	80	8.6	11.4	Moderate
	Paclitaxel (200 mg/m^2^) + Carboplatin (6 mg/ mL*min)	32	58	75	81.3	21.9	6.3	
Niho [26]	Paclitaxel (200 mg/m^2^) + Carboplatin (6 mg/mL*min) + Bevacizumab (15 mg/kg)	121	61	63.6	68.6	0	0	High
	Paclitaxel (200 mg/m^2^) + Carboplatin (6 mg/ mL*min)	59	60	64.4	71.2	0	0	
Patel [27]	Pemetrexed (500 mg/m^2^) + Carboplatin (6 mg/mL*min) + Bevacizumab (15 mg/kg)	472	65	53.2	89.8	0	0	High
	Paclitaxel (200 mg/m^2^) + Carboplatin (6 mg/ mL*min) + Bevacizumab (15 mg/kg)	467	65	53.3	89.9	0	0	
Pereira [28]	Pemetrexed (500 mg/m^2^) + Carboplatin (5 mg/ mL*min)	106	60	60.4	84.0	0	14.2	Moderate
	Docetaxel (75 mg/m^2^) + Carboplatin (5 mg/mL*min)	105	59	47.6	78.1	0	16.2	
Reck [29]	Gemcitabine (1,250 mg/m^2^) + Cisplatin (80 mg/m^2^) + Bevacizumab (15 mg/kg)	351	59	62.4	76.6	0	0	High
	Gemcitabine (1,250 mg/m^2^) + Cisplatin (80 mg/m^2^)	347	59	64.3	76.7	0	–	
Sandler [8]	Paclitaxel (200 mg/m^2^) + Carboplatin (6 mg/mL*min) + Bevacizumab (15 mg/kg)	417	56	50.4	74.3	0	0	High
	Paclitaxel (200 mg/m^2^) + Carboplatin (6 mg/ mL*min)	433	58	58.4	77.8	0	0	
Scagliotti [30]	Pemetrexed (500 mg/m^2^) + Cisplatin (75 mg/m^2^)	862	61	70.2	76.2	40.6	0	Moderate
	Gemcitabine (1,000 mg/m^2^) + Cisplatin (75 mg/m^2^)	863	61	75.7	75.7	43.5	0	
Scagliotti [31]	Gemcitabine (1,250 mg/m^2^) + Cisplatin (75 mg/m^2^)	205	63	81.5	81.5	32.7	5.4	Moderate
	Vinorelbine (25 mg/m^2^) + Cisplatin (75 mg/m^2^)	201	62	78.1	81.1	27.4	8.0	
	Paclitaxel (225 mg/m^2^) + Cisplatin (75 mg/m^2^)	201	62	75.6	81.6	32.3	8.5	
Schiller [32]	Gemcitabine (1,000 mg/m^2^) + Cisplatin (100 mg/m^2^)	288	64	62.0	89.0	22.3	6.0	High
	Paclitaxel (135 mg/m^2^) + Cisplatin (75 mg/m^2^)	288	62	64.0	86.0	26.8	5.0	
	Docetaxel (75 mg/m^2^) + Cisplatin (75 mg/m^2^)	289	63	62.0	86.0	25.0	6.0	
	Paclitaxel (225 mg/m^2^) + Carboplatin (6 mg/mL*min)	290	63	63.0	86.0	25.9	5.0	
Smit [33]	Gemcitabine (1,250 mg/m^2^) + Cisplatin (80 mg/m^2^)	160	57	70.6	78.8	25.6	11.3	High
	Paclitaxel (175 mg/m^2^) + Cisplatin (80 mg/m^2^)	159	57	59.7	81.8	18.9	11.9	
Soria [34]	Paclitaxel (200 mg/m^2^) + Carboplatin (6 mg/mL*min)	41	62	59	98	49	0	High
	Paclitaxel (200 mg/m^2^) + Carboplatin (6 mg/mL*min) + Bevacizumab (15 mg/kg)	44	58	52	95	0	0	
Spigel [35]	Ixabepilone (30 mg/m^2^) + Carboplatin (6 mg/mL*min)	42	63	57	69	47	0	High
	Ixabepilone (30 mg/m^2^) + Carboplatin (6 mg/mL*min) + Bevacizumab(15 mg/kg)	40	63	48	67	3	0	
Spigel [36]	Pemetrexed (500 mg/m^2^) + Carboplatin (5 mg/mL*min) + Bevacizumab (15 mg/kg)	61	73	56	97	0	100	Moderate
	Pemerexed (500 mg/m^2^)	48	72	63	90	0	100	
	Pemetrexed (500 mg/m^2^) + Bevacizumab (15 mg/kg)	63	72	57	92	0	100	
Treat [37]	Gemcitabine (1,000 mg/m^2^) + Carboplatin (75 mg/m^2^)	379	64.1	58.3	90	17.7	0.3	Moderate
	Paclitaxel (225 mg/m^2^) + Carboplatin (6 mg/ mL*min)	379	64.1	60.9	89.4	16.1	0.3	
Wu [38]	Pemetrexed (500 mg/m^2^) + Cisplatin (75 mg/m^2^)	126	57	56.3	84.9	0	0	High
	Gemcitabine (1,250 mg/m^2^) + Cisplatin (75 mg/m^2^)	130	56	54.6	84.6	0	0	
Zinner [39]	Pemetrexed (500 mg/m^2^) + Carboplatin (6 mg/mL*min)	182	66	57.5	99.5	0	0	High
	Paclitaxel (200 mg/m^2^) + Carboplatin (6 mg/ mL*min) + Bevacizumab (15 mg/kg)	179	65	58.1	100	0	0	
Zhao [40]	Endostar(7.5 mg/ m^2^ on days 1 to 14) + Gemcitabine (1000 mg/m^2^,days 1 and 8) + Cisplatin ( 30 mg/m^2^,day 2–4)	33	61	63.4	84.85	45.45	9.09	Moderate
	Gemcitabine (1000 mg/m^2^,days 1 and 8) + Cisplatin ( 30 mg/m^2^,day 2–4)	36	60	69.44	83.33	52.78	11.11	
Zhou [41]	Paclitaxel (175 mg/m^2^) + Carboplatin (6 mg/mL*min) + Bevacizumab (15 mg/kg)	138	57	54.3	91.3	0	0	High
	Paclitaxel (175 mg/m^2^) + Carboplatin (6 mg/ mL*min)	138	56	55.8	90.6	0	0	
Marinis [42]	Bevacizumab (7.5 mg/kg) + Gemcitabine (1,200 mg/m^2^)	44	74.2	62.8	90.7	0	0	High
	Bevacizumab (7.5 mg/k)g + Cisplatin (60 mg/m^2^) + Gemcitabine (1,000 mg/m^2^)	42	73.9	70	97.5	0	0	
Yu [43]	Gemcitabine (1000 mg/m^2^) + Carboplatin (5 mg/mL*min)	25	56.7	76	60	56	NA	High
	Gemcitabine (1000 mg/m^2^) + Carboplatin (5 mg/mL*min) + Endostar (7.5 mg/m^2^)	24	56.3	70.8	62.5	50	NA	
Schuette [44]	Bevacizumab (7.5 mg/kg) + Pemetrexed (500 mg/m^2^)	119	72.3	62.2	95	0	5	High
	Bevacizumab (7.5 mg/kg) + Pemetrexed (500 mg/m^2^) + Carboplatin (5 mg/mL*min)	134	71.9	64.2	95.5	0	5	
Zhou [45]	Pemetrexed (500 mg/m^2^) + Cisplatin (75 mg/m^2^) + Endostar (7.5 mg/m^2^)	56	54.1	51.8	87.5	0	0	Moderate
	Pemetrexed (500 mg/m^2^) + Cisplatin (75 mg/m^2^)	39	57.4	69.2	94.9	0	2.6	
Liu [46]	Vinorelbine (25 mg/m^2^) + Cisplatin (75 mg/m^2^) + Endostar (7.5 mg/m^2^)	19	55	57.9	57.o	36.8	5.3	Moderate
	Vinorelbine (25 mg/m^2^) + Cisplatin (75 mg/m^2^)	34	58.4	52.9	61.8	20.6	11.8	
Sun [47]	Endostar (7.5 mg/m^2^ on days 1 to 14) + Vinorelbine (25 mg/m^2^) + Cisplatin (75 mg/m^2^)	928	57.5	67.7	69.7	30.5	NA	High
	Endostar (7.5 mg/m^2^ on days 1 to 14) + Paclitaxel (150 mg/m^2^) + Cisplatin (75 mg/m^2^)	976	55.6	65.9	69.3	31.7	NA	
	Endostar (7.5 mg/m^2^ on days 1 to 14) + Gemcitabine (800 ~ 1000 mg/m^2^) + Cisplatin (75 mg/m^2^)	441	58.1	75.3	71.7	33.1	NA	
	Endostar (7.5 mg/m^2^ on days 1 to 14) + Gemcitabine (dose unavaliable) + cisplatin (dose unavaliable)	338	59.0	71.3	68.6	29.3	NA	

### Network meta-analysis of the outcome measures

**Fig. 2:**
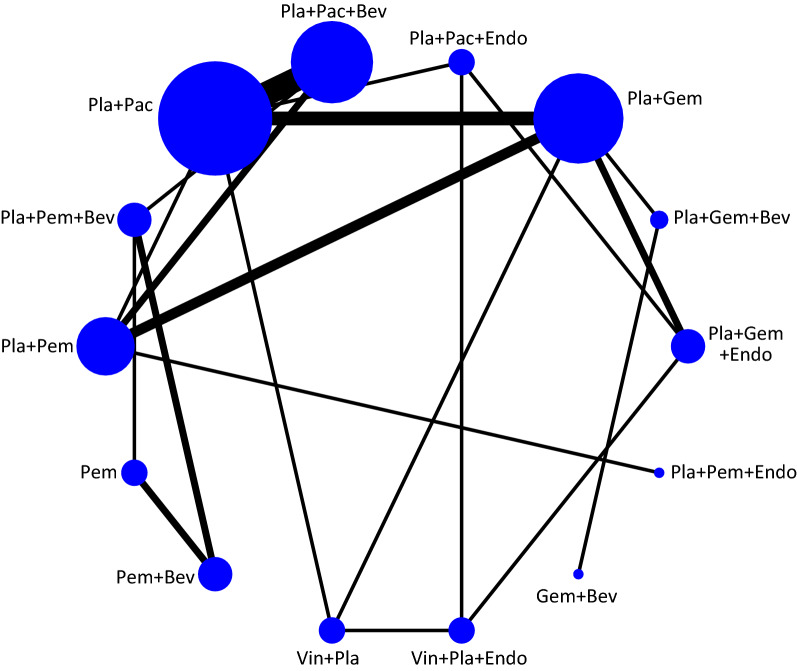
Network plot of 14 interventions for the treatment of NSCLC according to Bayesian network meta-analysis. Each node represents a treatment, and the size of the node is proportional to the number of patients. The width of the lines between two nodes represents the number of head‐to‐head trials

**Fig. 3:**
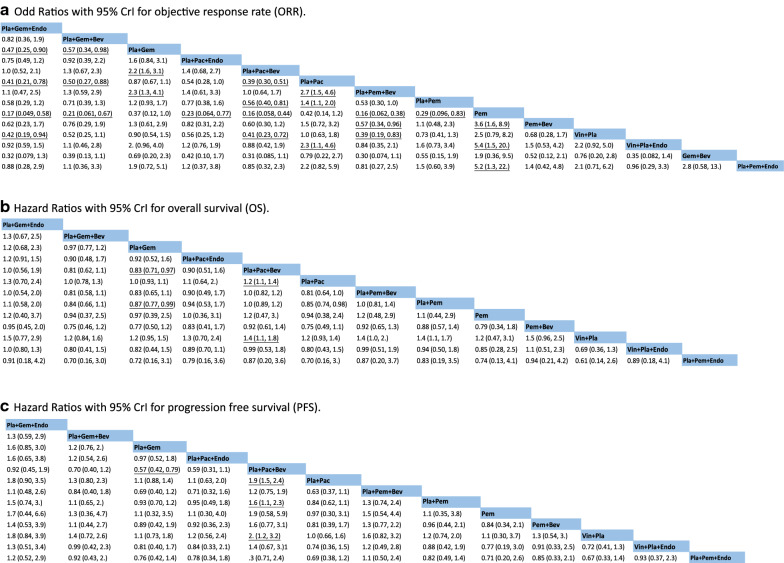
ORs or HRs between the included interventions according to the results of network meta-analysis (the treatment in the column compared with the treatment in the row)

To facilitate the study, we summarized the interventions involved in the articles as follows: platinum + gemcitabine + Endostar (Pla + Gem + Endo), platinum + gemcitabine + bevaczumab (Pla + Gem + Bev), platinum + gemcitabine (Pla + Gem), platinum + paclitaxel + Endostar (Pla + Pac + Endo), platinum + paclitaxel + bevacizumab (Pla + Pac + Bev), platinum + paclitaxel (Pla + Pac), platinum + pemetrexed + bevacizumab (Pla + Pem + Bev), platinum + pemetrexed (Pla + Pem), pemetrexed + bevacizumab (Pem + Bev), vinorelbine + platinum (Vin + Pla), vinorelbine + platinum + Endostar (Vin + Pla + Endo), gemcitabine + bevacizumab (Gem + Bev) and platinum + pemetrexed + Endostar (Pla + Pem + Endo). A total of 12,862 patients were treated with these interventions. The sample size of patients included in each intervention and the number of head-to-head comparisons were roughly described as a network plot, as shown in Fig. 2. The results of the Bayesian meta-analysis of ORR, OS and PFS are summarized in Fig. 3. The analyses of OS and PFS were not fully included for all interventions because some studies were unable to provide data related to ORR, OS or PFS.

For ORR, there were no significant differences (the CrI of the OR value included 1) in the improvement of the ORR of NSCLC patients between 4 different Endostar combined with chemotherapy regimens (Pla + Gem + Endo, Pla + Pac + Endo, and Vin + Pla + Endo, Pla + Pem + Endo) and 5 bevacizumab combined with chemotherapy regimens (Pla + Gem + Bev, Pla + Pac + Bev, Pla + Pem + Bev, Pem + Bev and Gem + Bev). There were no significant differences among the 4 Endostar combined with chemotherapy regimens. However, some Endostar combined with chemotherapy regimens had significant advantages over some chemotherapy regimens based on platinum. Pla + Gem + Endo was superior to Pla + Gem [OR: 0.47 (0.25, 0.90)], Pla + Pac [OR: 0.41 (0.21, 0.78)], and Vin + Pla [OR: 0.42 (0.19, 0.94)], and there were no significant differences between Pla + Pac + Endo, Vin + Pla + Endo and platinum-based chemotherapy, except Pla + Pac vs Vin + Pla + Endo [OR: 2.3 (1.1, 4.6)]. For the comparison among bevacizumab combined with chemotherapy regimens, only Pla + Pem + Bev was superior to Pem + Bev [OR: 0.57 (0.34, 0.96)]. Except for Pem + Bev and Gem + Bev, bevacizumab combined with chemotherapy was superior to some chemotherapy regimens: Pla + Gem + Bev was superior to Pla + Gem [OR: 0.57 (0.34, 0.98)] and Pla + Pac [OR: 0.50 (0.27, 0.88)]; Pla + Pac + Bev was better than Pla + Pac [OR: 0.39 (0.30, 0.51)], Pla + Pem [OR: 0.56 (0.40, 0.81)], and Vin + Pla [OR: 0.41 (0.23, 0.72)]; and Pla + Pem + Bev was better than Vin + Pla [OR: 0.39 (0.19, 0.83)]. Interestingly, for the improvement of ORR, Endostar combined with chemotherapy regimens (Pla + Gem + Endo, Pla + Pac + Endo, Vin + Pla + Endo and Pla + Pem + Endo) and bevacizumab combined with chemotherapy regimens (Pla + Gem + Bev, Pla + Pac + Bev, Pla + Pem + Bev and Pem + Bev), were significantly better than Pem alone, with ORs and 95% CrIs of 0.17 (0.049, 0.58), 0.21 (0.061, 0.67), 5.4 (1.5, 20.0), 5.2 (1.3, 22.0), 0.16 (0.058, 0.44), 0.23 (0.064, 0.77), and 0.16 (0.062, 0.38), 3.6 (1.6, 8.9), respectively.

For the time-to-event data of OS, there were no significant differences between the Endostar combined with chemotherapy regimens and the bevacizumab combined with chemotherapy regimens. For the bevacizumab combined with chemotherapy regimens, Pla + Pac + Bev was significantly better at prolonging OS than Pla + Gem [HR: 0.83 (0.71, 0.97)]; similarly, compared with Pla + Pac, Pla + Pac + Bev can also significantly reduce the HR [HR: 1.2 (1.1, 1.4)] (Pla + Pac vs Pla + Pac + Bev).

**Fig. 4:**
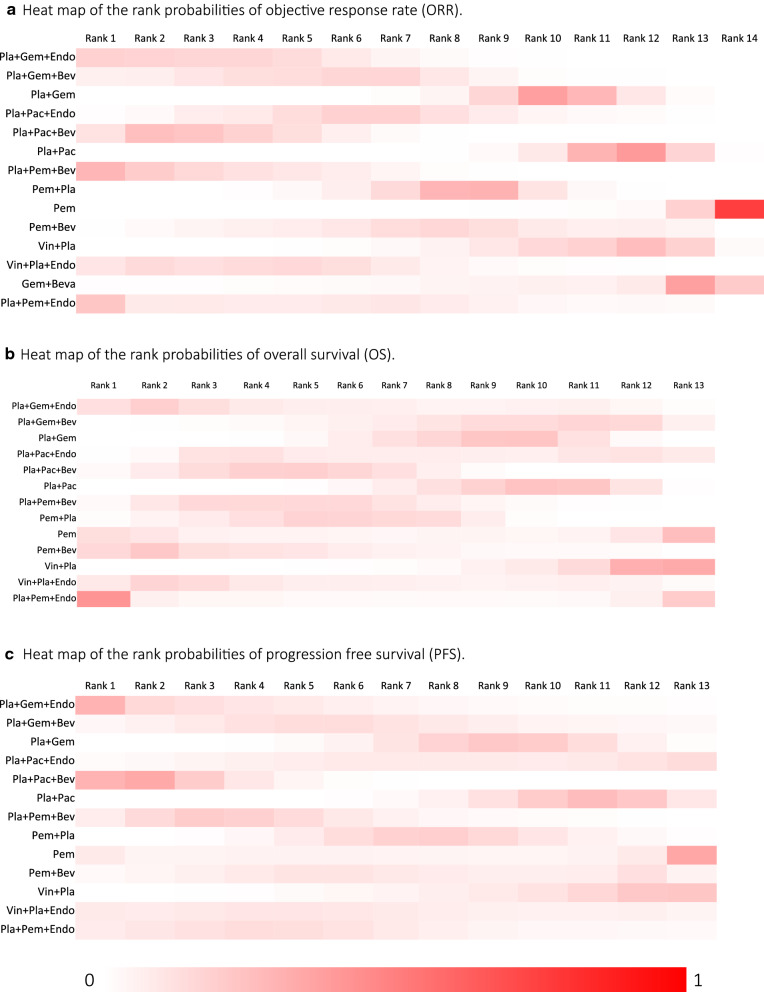
Heat map of the rank probabilities of each intervention according to the results of the network meta-analysis. Figure 3a: ORR. Figure 3b: OS. Figure 3c: PFS. The detailed data are provided in Additional file 1: Table S1

The results of rank probabilities for ORR, OS and PFS were shown as heat maps in Fig. 4 (See more detailed data in Additional file 1: Table S1). For the time-to-event data of PFS, there were no significant differences between the Endostar combined with chemotherapy regimens and the bevacizumab combined with chemotherapy regimens, similarly. And for the bevacizumab combined with chemotherapy regimens, Pla + Pac + Bev was superior to Pla + Gem [HR: 0.57 (0.42, 0.79)] and Pla + Pac [HR: 1.9 (1.5, 2.4)] (Pla + Pac vs Pla + Pac + Bev).

According to the results of network meta-analysis for ORR, Pla + Pem + Bev was the most likely best intervention (0.288) and Pla + Pac + Bev (0.255) was the most likely second best intervention. In terms of the time-to-event data of OS, Pla + Pem + Endo was the most likely best intervention (0.423), Pem + Bev (0.219) was the most likely second best intervention. And for the time-to-event data of PFS, the most likely best intervention was Pla + Gem + Endo (0.302), and the most likely second best intervention was Pla + Pac + Bev (0.340).

### Nodesplit and heterogeneity analyses

**Fig. 5:**
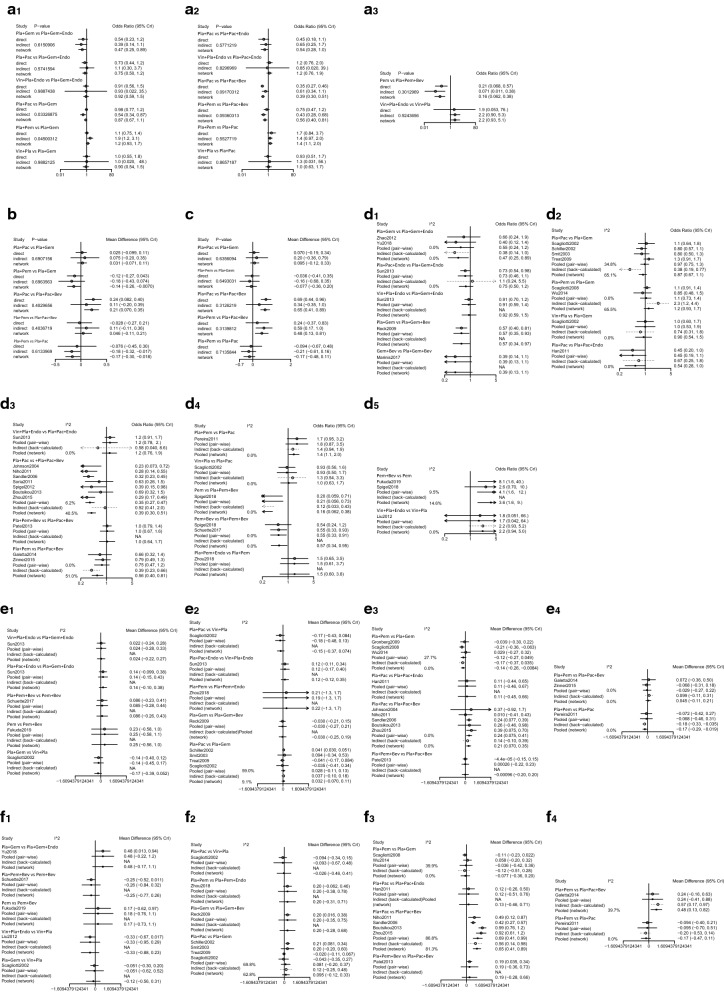
Nodesplit analysis and analysis of heterogeneity for ORR, OS and PFS. **a1** ~ **a**_**3**_ Nodesplit analysis of ORR. **b** Nodesplit analysis of OS. C: Nodesplit analysis of PFS. **d1** ~ **d**_**5**_ Analysis of heterogeneity for ORR. **e1** ~ **e**_**4**_ Analysis of heterogeneity for OS. **f1** ~ **f**_**4**_ Analysis of heterogeneity for PFS

The results of nodesplit analysis and heterogeneity were shown in Fig. 5. To test the consistency and heterogeneity between the network meta-analysis results and the head-to-head analysis results, we conducted nodesplit and heterogeneity analyses. For nodesplit analysis, in terms of ORR, the P values of Pla + Pac vs Pla + Gem and Pla + Pem vs Pla + Gem were significantly different, at 0.0333 and 0.0450, respectively, which indicated that the direct and indirect comparison results between Pla + Pac vs Pla + Gem and Pla + Pem vs Pla + Gem were inconsistent; for other comparisons of ORR and the nodesplit analysis results of OS and PFS, there were no significant differences. For the heterogeneity analysis, except for the high heterogeneity of Pla + Pac vs Pla + Pac + Bev (81.3%) for PFS, moderate heterogeneity of Pla + Pac vs Pla + Gem and for ORR (65.1%) and PFS (62.8%), moderate heterogeneity of Pla + Pem vs Pla + Gem (65.5%) and Pla + Pem vs Pla + Pac + Bev (51.0%) for ORR, there was small heterogeneity for the other results.

### Single-arm meta-analysis

**Fig. 6:**
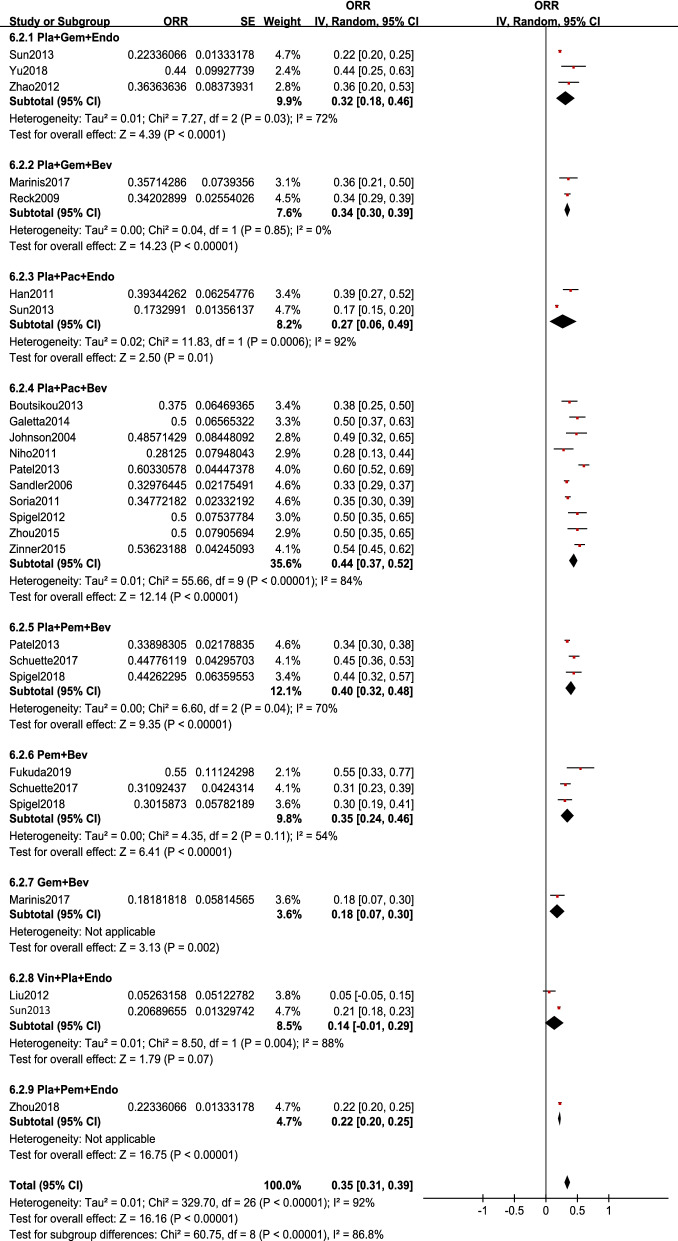
Single-arm meta-analysis of the ORR of patients treated with antiangiogenic agents combined with chemotherapy

**Fig. 7:**
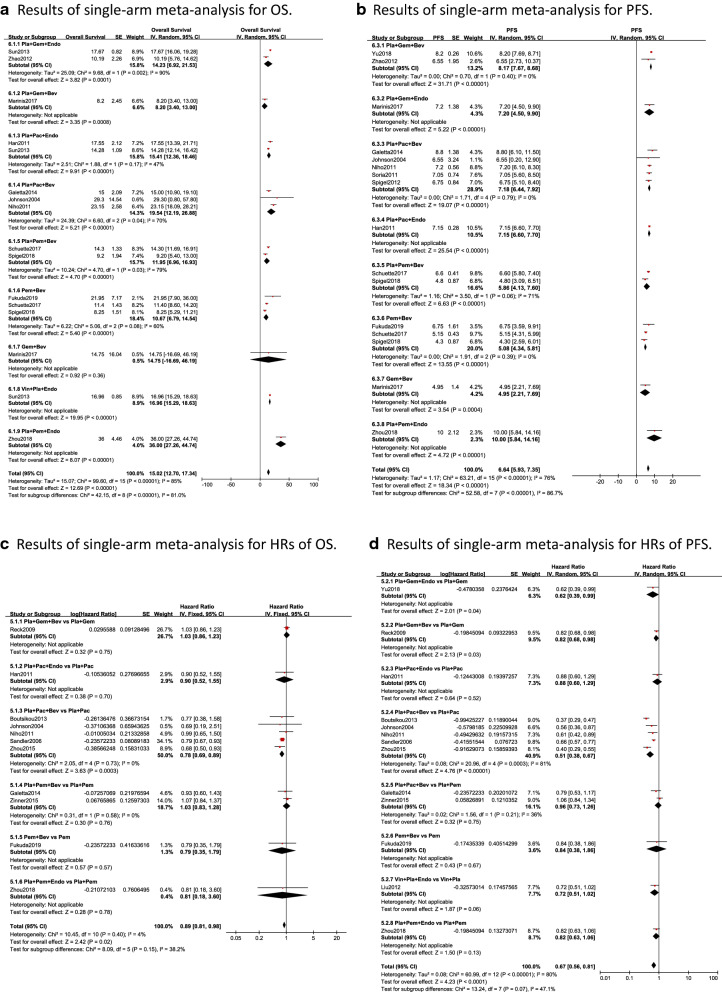
Single-arm meta-analysis of the OS and PFS of patients treated with antiangiogenic agents combined with chemotherapy

The forest plots of single-arm meta-analysis are shown in Fig. 6 (ORR) and Fig. 7 (OS and PFS). The forest plots of the event rate of adverse events are shown in Additional file 1: Figure S2.

1. ORR
  The pooled ORR was 0.35, and the 95% CI was 0.31, 0.39. Except for the synthesis result of Vin + Pla + Endo containing 0, the synthesis ORR values of Pla + Pac + Bev, Pla + Pem + Bev, were similar, which were 0.44 (0.37, 0.52), 0.40 (0.32, 0.48); followed by Pem + Bev and Pla + Gem + Bev and Pla + Gem + Endo, which were 0.35 (0.24, 0.46), 0.34 (0.30, 0.39) and 0.32(0.18, 0.46), respectively. And followed by Pla + Pac + Endo and Pla + Pem + Endo, with synthesis ORR values of 0.27 (0.06, 0.49) and 0.22 (0.20, 0.25), respectively. Gem + Bev was the worst intervention among them, with pooled ORR: 0.18(0.07, 0.30).
2. OS and PFS
  The pooled OS was 15.02 months, and the 95% CI was 12.70, 17.34. In addition to the result of Gem + Bev containing 0, Pla + Pem + Endo had the largest synthesis result: 36.00 months with a 95% CI of 12.70, 17.34; the second largest was that of Pla + Pac + Bev: 19.54 months with a 95% CI of 12.19, 26.88; followed by Vin + Pla + Endo, Pla + Pac + Endo and Pla + Gem + Endo, with synthesized values of 16.96 (15.29, 18.60), 15.41 (12.36, 18.46) and 14.23(6.92, 21.53), respectively; the worst synthesis result was Pla + Gem + Bev, with a result of 8.20 (3.40, 13.00). The pooled HR of OS was 0.89 (0.81, 0.98). The comparison between the included Endostar combined with chemotherapy regimens with chemotherapy regimens (Pla + Gem + Endo vs Pla + Gem, Pla + Pem + Endo vs Pla + Pem) had no statistical significance, with HR value and 95% CI 1.03(0.86, 1.23) and 0.81(0.18, 3.60), respectively; Among the bevacizumab combined with chemotherapy regimens vs chemotherapy regimens, only Pla + Pac + Bev vs Pla + Pac had statistical significance: 0.78 (0.69, 0.89); For PFS, the pooled HR and 95% CI were 0.67 (0.56, 0.81). Only Pla + Gem + Endo vs Pla + Gem was statistically siginificant among the results of comparison of Endostar combined with chemotherapy regimens with chemotherapy regimens, with HR value and 95% CI 0.62 (0.39, 0.99). For bevacizumab combined with chemotherapy vs chemotherapy regimens, Pla + Pac + Bev vs Pla + Pac and Pla + Gem + Bev vs Pla + Gem could significantly prolong PFS, with HR values of 0.51 (0.38, 0.67) and 0.82 (0.68, 0.98), respectively.
3. Event rate of adverse events
  Four most common adverse events between bevacizumab combined with chemotherapy and Endostar combined with chemotherapy, including anemia, thrombocytopenia, leukopenia and vomiting, were analysed in the current study. For anemia, the pooled event rate was 0.42 (0.29, 0.54). Except the result of Pla + Gem + Endo containing 0, the interventions with the highest incidence of anemia were Pem + Bev [0.58 (0.21, 0.96)] and Pla + Pem + Bev [0.55 (0.36, 0.74)], followed by Pla + Pac + Bev [0.32 (0.08, 0.57)] and Pla + Gem + Bev [0.30 (0.16, 0.44)], and the lowest was Gem + Bev [0.09 (0.01, 0.18)]. The pooled event rate of leukopenia was 0.57 (0.32, 0.82). Expect the result of Gem + Bev containing 0, the highest three were: Pla + Pac + Endo [0.87 (0.79, 0.95)], Pla + Pac + Bev [0.76 (0.39, 1.13)] and Pla + Gem + Endo [0.68 (0.27, 1.09)]; the intervention with the lowest incidence of leukopenia was Pla + Gem + Bev [0.28 (0.14, 0.41)]. For thrombocytopenia, the pooled event rate was 0.30 (0.15, 0.44). The intervention with the highest incidence of thrombocytopenia was Pla + Pem + Bev [0.48 (0.39, 0.57)]; the lowest was Pla + Pem + Endo [0.14 (0.05, 0.23)] and Gem + Bev [0.12 (0.02, 0.21)]. Finally, the pooled event rate of vomiting was 0.20 (0.11, 0.30). In addition to the result of Gem + Bev containing 0, Pla + Pem + Endo had the highest incidence [0.86 (0.77, 0.95)], while Pla + Pac + Endo [0.15 (0.06, 0.23)] and Pla + Gem + Bev [0.09 (0.06, 0.12)] had the lowest incidence of vomiting. For leukopenia and thrombocytopenia, potential publication bias may exist according to the results of Eegger’s test (p = 0.006 and p = 0.002, respectively). More details of the results of Begg’s and Egger’s test and funnel plots are shown in Additional file 1: Table S2 and Figure S3.

## Discussion

Angiogenesis plays an important role in tumorigenesis and development and is related to tumor proliferation, invasion and metastasis [4]. The results of clinical studies has suggested the combination of antiangiogenic agents and chemotherapy was effective, which can improve the ORR and prolong the OS and PFS for NSCLC patients. However, bevacizumab combined with chemotherapy and Endostar combined with chemotherapy are still lack of head-to-head clinical trials, which limits the clinical application of antiangiogenic agents combined with chemotherapy in the treatment of NSCLC. Therefore, by conducting the current Bayesian network meta-analysis, the effects of 14 interventions included in our study on improving ORR, prolonging PFS and OS were compared indirectly, so as to provide possible suggestions for further clinical trials and rational application of antiangiogenic agents and chemotherapy in the treatment of NSCLC. Our study showed that antiangiogenic agents combined with dual chemotherapy agents containing platinum may have a better effect on NSCLC patients. In terms of ORR, bevacizumab combined with chemotheray may have a better effect for the treatment of NSCLC; and in terms of OS and PFS, Endostar combined with chemotherapy may be superior to bevacizumab combined with chemotherapy.

The results of the single-arm meta-analysis showed that the combination of antiangiogenic agents and chemotherapy agents could improve ORR [pooled ORR and 95% CI: 0.35 (0.31, 0.39)], OS [pooled HR and 95% CI: 0.89 (0.81, 0.98)] and PFS [pooled HR and 95% CI: 0.67 (0.56, 0.81)], which showed that antiangiogenic agents combined with chemotherapy was more effective than chemotherapy alone. However, at present, it is still unclear that antiangiogenic agents in combination with which chemotherapy regimen can provide more benefits to patients in the treatment of NSCLC. Based on the first-ranked and second-ranked interventions of rank probabilities, our analysis found that, in terms of ORR, antiangiogenic agents combined with Pla + Pem or Pla + Pac may have better therapeutic effects; in terms of OS, antiangiogenic agents combined with Pla + Pem or Pem may be superior to prolonging OS and in terms of PFS, combined with Pla + Gem or Pla + Pac may have better effects. Therefore, our results showed than antiangiogenic agents combined with Pla + Pem, Pla + Pac or Pla + Pem may benefit patients more in the treatment of NSCLC. Among them, antiangiogenic agents combined with Pla + Pem seems to be more conducive to improving ORR and prolonging OS and combined with Pla + Pac seems to be the first choice for prolonging OS and PFS.

The combinations of Endostar, an antiangiogenic agent, combined with chemotherapy and bevacizumab combined with chemotherapy have shown good results. However, the comparison between Endostar combined with  chemotherapy and bevacizumab combined with chemotherapy lacks head-to-head clinical trials. Our results suggested that there was no significant difference in the effect of improving ORR and prolonging OS and PFS in NSCLC patients between Endostar combined with chemotherapy and bevacizumab combined with chemotherapy. Although there were no significant differences between these interventions, the rank positions indicated that Pla + Pem + Bev and Pla + Pac + Bev were in a more advanced position for improving ORR, and in terms of OS, Pla + Gem + Endo may be the best choice among these interventions. And interesting, for prolnging PFS, our results showed that Pla + Pem + Endo may be the first-ranked regimen, however, as the only bevacizumab combined with chemotherapy regimen for the treatment of non-squamous NSCLC aproved by FDA, Pla + Pac + Bev was the second-ranked regimen for improving PFS. Our analysis indicaded that bevacizumab combined with chemotherapy seems more effective for improving ORR and Endostar seems more effective for prolonging OS and PFS.

For the comparison of the different Endostar combined with chemotherapy regimens, a 4-phase clinical study showed that there were no significant differences in improving the ORR, OS and TTP (time to progression) among Vin + Pla + Endo, Pla + Gem + Endo and Pla + Pac + Endo [47]. However, for Pla + Pem + Endo, there was few head-to-head comparisons with other Endostar combined chemotherapy regimens. Our network meta-analysis showed that compared with Vin + Pla + Endo, Pla + Gem + Endo and Pla + Pac + Endo, there were no statistical difference in improving ORR and prolonging OS and PFS. However, the results of single-arm meta-analysis showed that some differences still existed among them. Compared with Pla + Gem + Endo, the ORR [ 0.22 (0.20, 0.25) vs 0.32 (0.18, 0.46)], OS [ 36.00 months (27.26, 44.74) vs 14.23 months (6.92, 21.23)] and PFS [10.00 months (5.84, 14.16) vs 8.17 months (7.67, 8.68)] of Pla + Pem + Endo were obviously lower or longer. Similarly, compared with Pla + Pac + Endo, the OS [ 36.00 months (27.26, 44.74) vs 15.41 months (12.36, 18.46)] and PFS [10.00 months (5.84, 14.16) vs 7.15 months (6.60, 7.70)] of Pla + Pem + Endo were evidently longer, although there was no obvious difference for ORR [ 0.22 (0.20, 0.25) vs 0.27 (0.06, 0.49)]. The reason may be related to the differences and inconsistencies between the studies and the limited number of studies. In the future, head-to-head randomized controlled trials are needed to determine the relationship between the above strategies for improving the prognosis of patients to provide more reasonable treatments for NSCLC patients.

For adverse events, the event rate of leukopenia [0.57 (0.32, 0.82)] was the highest among the four most common adverse events between Endostar combined with chemotherapy and bevacizumab combined with chemotherapy. It is worth noting that the common adverse events of bevacizumab combined with chemotherapy, such as hypertension, proteinuria and thromboembolism, have not been studied in the study of Endostar combined with chemotherapy. Compared with bevacizumab combined with chemotherapy, in Pla + Pac + Endo, the event rate of leukopenia was the highest [0.87 (0.79, 0.95)], while the incidence of vomiting was the second lowest [0.15 (0.06, 0.23)]. In addition, in Pla + Pem + Endo, the event rate of vomiting was the highest [0.86 (0.77, 0.95)] and the incidence of thrombocytopenia was the second lowest [0.14 (0.05, 0.23)]. Gem + Bev had a low incidence in both anemia [0.09 (0.01, 0.18)] and thrombocytopenia [0.12 (0.02, 0.21)]. However, due to the high heterogeneity, limited number and potential publication bias of studies, further studies are needed to confirm these conclusions.

For our network meta-analysis, there were still some limitations. First, we included 11 moderate-quality studies and 18 high-quality studies: of the 25 randomized controlled trials, 10 were moderate-quality articles and 15 were high-quality articles; the other 4 were nonrandomized controlled trials and prospective studies, of which 3 were high-quality articles and 1 was a moderate quality article according to the NOS. Although the number of included nonrandomized controlled trials and prospective studies was relatively small, the methodological design of the randomized clinical trials was still more reliable than that of the nonrandomized controlled trials, which may lead to inconsistency in our results. Second, for the patients who participated in the study, squamous cell carcinoma and non-squamous cell carcinoma were not distinguished, which may have some impact on the consistency of our results. For squamous cell carcinoma, there were no independent study data and thus, we failed to observe the curative effect of Endostar combined with chemotherapy and bevacizumab combined with chemotherapy in squamous cell carcinoma. Third, in order to facilitate the analysis, we did not make a strict distinction between the dosage and the method of administration. In addition, original data and laboratory data was lacking in the current meta-analysis. Finally, our results were based on a Bayesian network meta-analysis and the statistical analysis of various interventions; however, the performance of various interventions in real patients still needs to be confirmed by head-to-head clinical trials.

## Conclusions

In summary, the combination of antiangiogenic agents with platinum-containing dual drug chemotherapy can improve NSCLC patients’ benefit. The combination of Endostar and platinum-containing dual drugs may be a better choice to prolong OS and PFS. More clincial trials are needed to ensure the reasonable use of antiangiogenic combined with chemotheray regimens for NSCLC patients in the future.

## Supplementary information

**Additional file 1: Table S1.** Rank probabilities of each treatment for different outcome measures based on the network meta-analysis.** Table S2.** Begg's and Egger's tests of the single-arm meta-analysis.** Table S3.** PRISMA checklist of the current network meta-analysis.** Figure S1.** The details of quality assessment of included studies. A. The details of quality assessment using the Cochrane Collaboration's risk of bias tool for 25 randomized controlled trials. B. The details of quality assessment using Newcastle Ottawa Scale for 4 nonrandomized controlled trials.** Figure S2.** Results of single-arm meta-analysis of four common adverse events of bevacizumab combined with chemotherapy and Endostar combined with hemotherapy. A. Anemia; B. Leukopenia. C. Thrombocytopenia. D. Vomiting.** Figure S3. **Funnel plots of the single-arm meta-analyisis. A: ORR. B: OS. C: PFS. D: HR of OS. E: HR of PFS. F: Anemia. G: Leukopenia. H: Thrombocytopenia. I: Vomiting.

## Data Availability

The datasets analyzed during the current study are available from the corresponding author on reasonable request.

## Abbreviations

NSCLC: Non-small cell lung cancer
VEGF: Vascular endothelial growth factor
PDGF: Platelet-derived growth factor
TGF-β: Transforming growth factor-β
FGF: Fibroblast growth factor
ORR: Objective response rate
PFS: Progression-free survival
OS: Overall survival
TTP: Time to progression
RCT: Randomized controlled trial
NOS: Newcastle ottawa scale
OR: Odd ratio
HR: Hazard ratio
CI: Confidence intervals
CrI: Credible intervals
Pla + Gem + Endo: Platinum + gemcitabine + Endostar
Pla + Gem + Bev: Platinum + gemcitabine + bevaczumab
Pla + Gem: Platinum + gemcitabine
Pla + Pac + Endo: Platinum + paclitaxel + Endostar
Pla + Pac + Bev: Platinum + paclitaxel + bevacizumab
Pla + Pac: Platinum + paclitaxel
Pla + Pem + Bev: Platinum + pemetrexed + bevacizumab
Pla + Pem: Platinum + pemetrexed
Pem + Bev: Pemetrexed + bevacizumab
Vin + Pla: Vinorelbine + platinum
Vin + Pla + Endo: Vinorelbine + platinum + Endostar
Gem + Bev: Gemcitabine + bevacizumab
Pla + Pem + Endo: Platinum + pemetrexed + Endostar
