# Supplementary material for: A comparison of the efficacy of antiangiogenic agents combined with chemotherapy for the treatment of non-small cell lung cancer: a network meta-analysis
Source: Cancer Cell Int. 2020 Nov 10;20:548. doi: 10.1186/s12935-020-01639-4 (PMC7653849; doi:10.1186/s12935-020-01639-4)
Supplement: Supplementary file 1 — Additional file 1: Table S1. Rank probabilities of each treatment for different outcome measures based on the network meta-analysis. Table S2. Begg's and Egger's tests of the single-arm meta-analysis. Table S3. PRISMA checklist of the current network meta-analysis. Figure S1. The details of quality assessment of included studies. A. The details of quality assessment using the Cochrane Collaboration's risk of bias tool for 25 randomized controlled trials. B. The details of quality assessment using Newcastle Ottawa Scale for 4 nonrandomized controlled trials. Figure S2. Results of single-arm meta-analysis of four common adverse events of bevacizumab combined with chemotherapy and Endostar combined with hemotherapy. A. Anemia; B. Leukopenia. C. Thrombocytopenia. D. Vomiting. Figure S3. Funnel plots of the single-arm meta-analyisis. A: ORR. B: OS. C: PFS. D: HR of OS. E: HR of PFS. F: Anemia. G: Leukopenia. H: Thrombocytopenia. I: Vomiting. [file 12935_2020_1639_MOESM1_ESM.pdf]

Table S1. Rank probabilities of each treatment for different outcome measures based on the network meta-analysis.

Table S2. Begg's and Egger's tests of the single-arm meta-analysis.

Table S3. PRISMA checklist of the current network meta-analysis.

Figure S1. The details of quality assessment of included studies. A. The details of quality assessment using the Cochrane Collaboration's risk of bias tool for 25 randomized controlled trials. B. The details of quality assessment using Newcastle Ottawa Scale for 4 nonrandomized controlled trials.

Figure S2. Results of single-arm meta-analysis of four common adverse events of bevacizumab combined with chemotherapy and Endostar combined with chemotherapy. A. Anemia; B. Leukopenia. C. Thrombocytopenia. D. Vomiting.

Figure S3. Funnel plots of the single-arm meta-analyisis. A: ORR. B: OS. C: PFS. D: HR of OS. E: HR of PFS. F: Anemia. G: Leukopenia. H: Thrombocytopenia. I: Vomiting.

Table S1

	Rank 1	Rank 2	Rank 3	Rank 4	Rank 5	Rank 6	Rank 7	Rank 8	Rank 9	Rank 10	Rank 11	Rank 12	Rank 13	Rank 14
ORR														
Pla+Gem+Endo	0.184	0.176	0.162	0.162	0.139	0.090	0.046	0.023	0.010	0.005	0.002	0.001	0.000	0.000
Pla+Gem+Bev	0.062	0.069	0.104	0.132	0.135	0.161	0.166	0.098	0.044	0.015	0.008	0.005	0.001	0.000
Pla+Gem	0.000	0.000	0.000	0.000	0.001	0.004	0.013	0.045	0.163	0.379	0.283	0.097	0.016	0.001
Pla+Pac+Endo	0.009	0.026	0.076	0.090	0.136	0.182	0.182	0.127	0.082	0.041	0.025	0.016	0.008	0.001
Pla+Pac+Bev	0.112	0.255	0.230	0.180	0.130	0.065	0.022	0.004	0.000	0.000	0.000	0.000	0.000	0.000
Pla+Pac	0.000	0.000	0.000	0.000	0.000	0.000	0.001	0.006	0.024	0.093	0.297	0.399	0.172	0.008
Pla+Pem+Bev	0.288	0.203	0.155	0.120	0.098	0.073	0.040	0.014	0.005	0.002	0.001	0.000	0.000	0.000
Pla+Pem	0.000	0.000	0.002	0.011	0.029	0.068	0.149	0.294	0.303	0.106	0.028	0.007	0.002	0.000
Pem	0.000	0.000	0.000	0.000	0.001	0.001	0.002	0.004	0.006	0.010	0.013	0.026	0.184	0.753
Pem+Bev	0.006	0.027	0.046	0.061	0.074	0.097	0.135	0.159	0.133	0.085	0.066	0.068	0.045	0.000
Vin+Pla	0.000	0.000	0.002	0.003	0.007	0.014	0.028	0.054	0.096	0.152	0.184	0.261	0.177	0.022
Vin+Pla+Endo	0.104	0.149	0.126	0.144	0.153	0.131	0.086	0.048	0.027	0.014	0.010	0.006	0.002	0.000
Gem+Bev	0.009	0.009	0.010	0.013	0.018	0.022	0.031	0.045	0.053	0.062	0.059	0.085	0.373	0.212
Pla+Pem+Endo	0.226	0.084	0.087	0.082	0.081	0.091	0.099	0.081	0.055	0.035	0.025	0.029	0.021	0.003
OS														
Pla+Gem+Endo	0.129	0.190	0.133	0.085	0.067	0.069	0.064	0.047	0.048	0.059	0.060	0.035	0.014	
Pla+Gem+Bev	0.003	0.007	0.014	0.022	0.039	0.061	0.080	0.108	0.142	0.144	0.165	0.154	0.062	
Pla+Gem	0.000	0.000	0.002	0.008	0.030	0.073	0.122	0.163	0.227	0.228	0.118	0.027	0.003	
Pla+Pac+Endo	0.005	0.025	0.113	0.121	0.081	0.071	0.071	0.078	0.070	0.066	0.099	0.114	0.085	
Pla+Pac+Bev	0.026	0.079	0.140	0.188	0.192	0.163	0.126	0.064	0.018	0.003	0.001	0.000	0.000	
Pla+Pac	0.000	0.000	0.001	0.002	0.010	0.033	0.073	0.122	0.178	0.239	0.224	0.109	0.010	
Pla+Pem+Bev	0.026	0.096	0.149	0.155	0.151	0.149	0.116	0.076	0.043	0.020	0.012	0.006	0.001	
Pla+Pem	0.014	0.048	0.079	0.127	0.176	0.170	0.155	0.143	0.071	0.014	0.003	0.001	0.000	

Pem	0.133	0.105	0.055	0.048	0.047	0.042	0.035	0.043	0.043	0.036	0.050	0.104	0.257
Pem+Bev	0.149	0.219	0.133	0.113	0.106	0.071	0.058	0.049	0.030	0.024	0.025	0.019	0.004
Vin+Pla	0.000	0.001	0.001	0.002	0.004	0.008	0.015	0.027	0.057	0.086	0.149	0.317	0.333
Vin+Pla+Endo	0.091	0.170	0.142	0.091	0.070	0.067	0.063	0.056	0.050	0.059	0.065	0.053	0.023
Pla+Pem+Endo	0.423	0.060	0.038	0.038	0.027	0.024	0.023	0.024	0.024	0.022	0.030	0.060	0.207
PFS													
Pla+Gem+Endo	0.302	0.155	0.127	0.104	0.088	0.064	0.048	0.033	0.024	0.018	0.014	0.013	0.012
Pla+Gem+Bev	0.032	0.058	0.090	0.118	0.135	0.135	0.117	0.091	0.068	0.050	0.042	0.034	0.028
Pla+Gem	0.000	0.000	0.001	0.006	0.021	0.053	0.108	0.176	0.219	0.207	0.137	0.058	0.014
Pla+Pac+Endo	0.017	0.029	0.045	0.062	0.075	0.088	0.090	0.086	0.083	0.081	0.094	0.111	0.138
Pla+Pac+Bev	0.301	0.340	0.202	0.097	0.039	0.014	0.005	0.002	0.000	0.000	0.000	0.000	0.000
Pla+Pac	0.000	0.000	0.000	0.001	0.003	0.009	0.025	0.056	0.118	0.206	0.264	0.222	0.096
Pla+Pem+Bev	0.074	0.146	0.201	0.188	0.143	0.092	0.058	0.034	0.025	0.017	0.015	0.005	0.001
Pla+Pem	0.000	0.002	0.010	0.035	0.082	0.137	0.182	0.192	0.155	0.105	0.058	0.031	0.011
Pem	0.086	0.044	0.045	0.052	0.053	0.054	0.051	0.044	0.040	0.043	0.053	0.086	0.350
Pem+Bev	0.024	0.042	0.063	0.089	0.107	0.107	0.093	0.079	0.069	0.068	0.076	0.129	0.054
Vin+Pla	0.000	0.002	0.005	0.011	0.021	0.036	0.049	0.069	0.088	0.114	0.159	0.219	0.225
Vin+Pla+Endo	0.085	0.082	0.092	0.103	0.102	0.096	0.084	0.072	0.063	0.057	0.057	0.062	0.046
Pla+Pem+Endo	0.079	0.100	0.121	0.134	0.130	0.115	0.090	0.066	0.046	0.035	0.031	0.028	0.024

Table S2

Results of Begg's and Egger's tests.

	Begg's test	Egger's test
ORR	0.441	0.000
OS	0.685	0.997
PFS	0.964	0.275
OS-HR	0.436	0.731
PFS-HR	0.760	0.890
Leukopenia	0.118	0.002
Thrombocytopenia	0.021	0.006
Anemia	1.000	0.236
Vomiting	0.697	0.561

Table S3

Section/topic	#	Checklist item	Reported on page #
**TITLE**			
Title	1	Identify the report as a systematic review, meta-analysis, or both.	1
**ABSTRACT**			
Structured summary	2	Provide a structured summary including, as applicable: background; objectives; data sources; study eligibility criteria, participants, and interventions; study appraisal and synthesis methods; results; limitations; conclusions and implications of key findings; systematic review registration number.	2, 3
**INTRODUCTION**			
Rationale	3	Describe the rationale for the review in the context of what is already known.	3, 4, 5
Objectives	4	Provide an explicit statement of questions being addressed with reference to participants, interventions, comparisons, outcomes, and study design (PICOS).	3, 4, 5
**METHODS**			
Protocol and registration	5	Indicate if a review protocol exists, if and where it can be accessed (e.g., Web address), and, if available, provide registration information including registration number.	NA
Eligibility criteria	6	Specify study characteristics (e.g., PICOS, length of follow-up) and report characteristics (e.g., years considered, language, publication status) used as criteria for eligibility, giving rationale.	6, 7
Information sources	7	Describe all information sources (e.g., databases with dates of coverage, contact with study authors to identify additional studies) in the search and date last searched.	6, 7
Search	8	Present full electronic search strategy for at least one database, including any limits used, such that it could be repeated.	5
Study selection	9	State the process for selecting studies (i.e., screening, eligibility, included in systematic review, and, if applicable, included in the meta-analysis).	5, 6
Data collection process	10	Describe method of data extraction from reports (e.g., piloted forms, independently, in duplicate) and any processes for obtaining and confirming data from investigators.	6
Data items	11	List and define all variables for which data were sought (e.g., PICOS, funding sources) and any assumptions and simplifications made.	7, 8
Risk of bias in individual studies	12	Describe methods used for assessing risk of bias of individual studies (including specification of whether this was done at the study or outcome level), and how this information is to be used in any data synthesis.	6
Summary measures	13	State the principal summary measures (e.g., risk ratio, difference in means).	6
Synthesis of results	14	Describe the methods of handling data and combining results of studies, if done, including measures of consistency (e.g., I^2) for each meta-analysis.	7, 8

Section/topic	#	Checklist item	Reported on page #
Risk of bias across studies	15	Specify any assessment of risk of bias that may affect the cumulative evidence (e.g., publication bias, selective reporting within studies).	Appendix
Additional analyses	16	Describe methods of additional analyses (e.g., sensitivity or subgroup analyses, meta-regression), if done, indicating which were pre-specified.	7, 8
**RESULTS**			
Study selection	17	Give numbers of studies screened, assessed for eligibility, and included in the review, with reasons for exclusions at each stage, ideally with a flow diagram.	8
Study characteristics	18	For each study, present characteristics for which data were extracted (e.g., study size, PICOS, follow-up period) and provide the citations.	8
Risk of bias within studies	19	Present data on risk of bias of each study and, if available, any outcome level assessment (see item 12).	Appendix
Results of individual studies	20	For all outcomes considered (benefits or harms), present, for each study: (a) simple summary data for each intervention group (b) effect estimates and confidence intervals, ideally with a forest plot.	8-10
Synthesis of results	21	Present results of each meta-analysis done, including confidence intervals and measures of consistency.	11-12
Risk of bias across studies	22	Present results of any assessment of risk of bias across studies (see Item 15).	Appendix
Additional analysis	23	Give results of additional analyses, if done (e.g., sensitivity or subgroup analyses, meta-regression [see Item 16]).	11, Appendix
**DISCUSSION**			
Summary of evidence	24	Summarize the main findings including the strength of evidence for each main outcome; consider their relevance to key groups (e.g., healthcare providers, users, and policy makers).	13-16
Limitations	25	Discuss limitations at study and outcome level (e.g., risk of bias), and at review-level (e.g., incomplete retrieval of identified research, reporting bias).	16-17
Conclusions	26	Provide a general interpretation of the results in the context of other evidence, and implications for future research.	17
**FUNDING**			
Funding	27	Describe sources of funding for the systematic review and other support (e.g., supply of data); role of funders for the systematic review.	NA

Figure S1

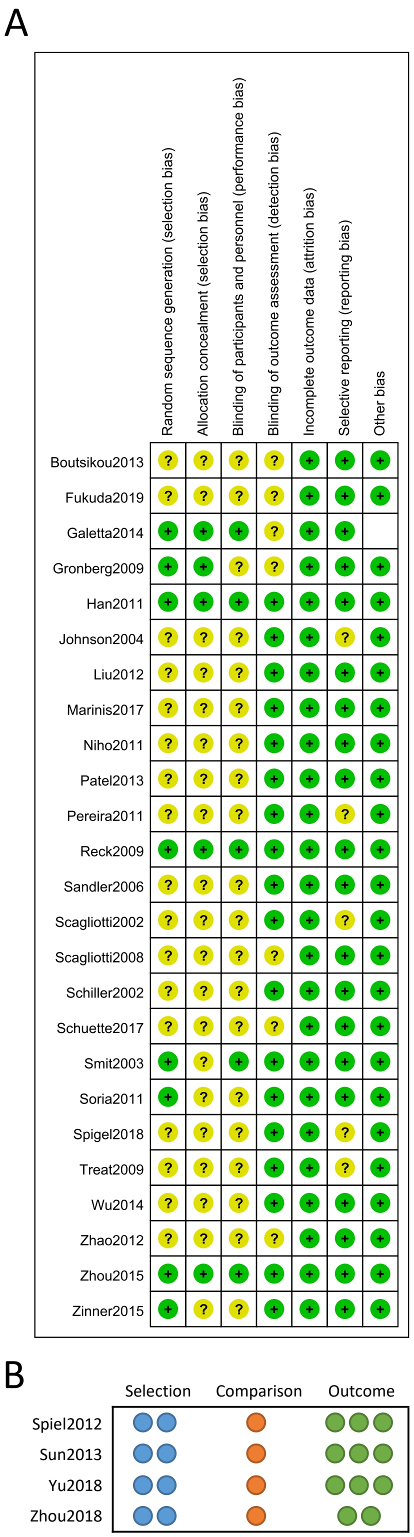
A
Random sequence generation (selection bias)
Allocation concealment (selection bias)
Blinding of participants and personnel (performance bias)
Blinding of outcome assessment (detection bias)
Incomplete outcome data (attrition bias)
Selective reporting (reporting bias)
Other bias
Boutsikou2013
Fukuda2019
Galetta2014
Gronberg2009
Han2011
Johnson2004
Liu2012
Marinis2017
Niho2011
Patel2013
Pereira2011
Reck2009
Sandler2006
Scagliotti2002
Scagliotti2008
Schiller2002
Schuette2017
Smit2003
Soria2011
Spigel2018
Treat2009
Wu2014
Zhao2012
Zhou2015
Zinner2015
B
Selection
Comparison
Outcome
Spiel2012
Sun2013
Yu2018
Zhou2018

Figure S2

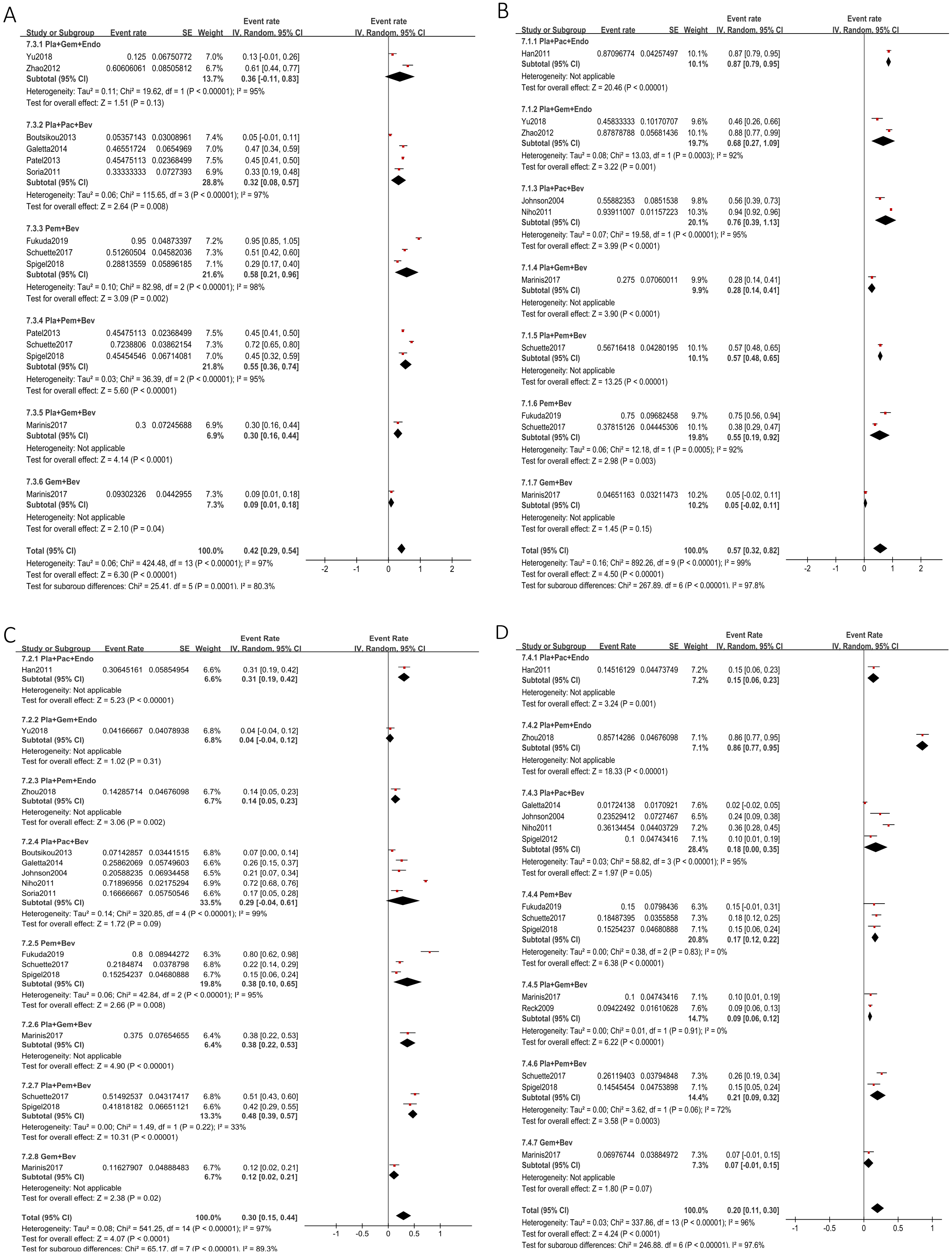
A
Study or Subgroup Event rate SE Weight Event rate IV, Random, 95% CI Event rate IV, Random, 95% CI
7.3.1 Pla+Gem+Endo
Yu2018 0.125 0.06750772 7.0% 0.13 [-0.01, 0.26]
Zhao2012 0.60606061 0.08505812 6.7% 0.61 [0.44, 0.77]
Subtotal (95% CI) 13.7% 0.36 [-0.11, 0.83]
Heterogeneity: Tau² = 0.11; Chi² = 19.62, df = 1 (P < 0.00001); I² = 95%
Test for overall effect: Z = 1.51 (P = 0.13)
7.3.2 Pla+Pac+Bev
Boutsikou2013 0.05357143 0.03008961 7.4% 0.05 [-0.01, 0.11]
Galetta2014 0.46551724 0.0654969 7.0% 0.47 [0.34, 0.59]
Patel2013 0.45475113 0.02368499 7.5% 0.45 [0.41, 0.50]
Soria2011 0.33333333 0.0727393 6.9% 0.33 [0.19, 0.48]
Subtotal (95% CI) 28.8% 0.32 [0.08, 0.57]
Heterogeneity: Tau² = 0.06; Chi² = 115.65, df = 3 (P < 0.00001); I² = 97%
Test for overall effect: Z = 2.64 (P = 0.008)
7.3.3 Pem+Bev
Fukuda2019 0.95 0.04873397 7.2% 0.95 [0.85, 1.05]
Schuette2017 0.51260504 0.04582036 7.3% 0.51 [0.42, 0.60]
Spigel2018 0.28813559 0.05896185 7.1% 0.29 [0.17, 0.40]
Subtotal (95% CI) 21.6% 0.58 [0.21, 0.96]
Heterogeneity: Tau² = 0.10; Chi² = 82.98, df = 2 (P < 0.00001); I² = 98%
Test for overall effect: Z = 3.09 (P = 0.002)
7.3.4 Pla+Pem+Bev
Patel2013 0.45475113 0.02368499 7.5% 0.45 [0.41, 0.50]
Schuette2017 0.7238806 0.03862154 7.3% 0.72 [0.65, 0.80]
Spigel2018 0.45454546 0.06714081 7.0% 0.45 [0.32, 0.59]
Subtotal (95% CI) 21.8% 0.55 [0.36, 0.74]
Heterogeneity: Tau² = 0.03; Chi² = 36.39, df = 2 (P < 0.00001); I² = 95%
Test for overall effect: Z = 5.60 (P < 0.00001)
7.3.5 Pla+Gem+Bev
Marinis2017 0.3 0.07245688 6.9% 0.30 [0.16, 0.44]
Subtotal (95% CI) 6.9% 0.30 [0.16, 0.44]
Heterogeneity: Not applicable
Test for overall effect: Z = 4.14 (P < 0.0001)
7.3.6 Gem+Bev
Marinis2017 0.09302326 0.0442955 7.3% 0.09 [0.01, 0.18]
Subtotal (95% CI) 7.3% 0.09 [0.01, 0.18]
Heterogeneity: Not applicable
Test for overall effect: Z = 2.10 (P = 0.04)
Total (95% CI) 100.0% 0.42 [0.29, 0.54]
Heterogeneity: Tau² = 0.06; Chi² = 424.48, df = 13 (P < 0.00001); I² = 97%
Test for overall effect: Z = 6.30 (P < 0.00001)
Test for subgroup differences: Chi² = 25.41, df = 5 (P = 0.0001), I² = 80.3%
-2 -1 0 1 2
B
Study or Subgroup Event rate SE Weight Event rate IV, Random, 95% CI Event rate IV, Random, 95% CI
7.1.1 Pla+Pac+Endo
Han2011 0.87096774 0.04257497 10.1% 0.87 [0.79, 0.95]
Subtotal (95% CI) 10.1% 0.87 [0.79, 0.95]
Heterogeneity: Not applicable
Test for overall effect: Z = 20.46 (P < 0.00001)
7.1.2 Pla+Gem+Endo
Yu2018 0.45833333 0.10170707 9.6% 0.46 [0.26, 0.66]
Zhao2012 0.87878788 0.05681436 10.1% 0.88 [0.77, 0.99]
Subtotal (95% CI) 19.7% 0.68 [0.27, 1.09]
Heterogeneity: Tau² = 0.08; Chi² = 13.03, df = 1 (P = 0.0003); I² = 92%
Test for overall effect: Z = 3.22 (P = 0.001)
7.1.3 Pla+Pac+Bev
Johnson2004 0.55882353 0.0851538 9.8% 0.56 [0.39, 0.73]
Niho2011 0.93911007 0.01157223 10.3% 0.94 [0.92, 0.96]
Subtotal (95% CI) 20.1% 0.76 [0.39, 1.13]
Heterogeneity: Tau² = 0.07; Chi² = 19.58, df = 1 (P < 0.00001); I² = 95%
Test for overall effect: Z = 3.99 (P < 0.0001)
7.1.4 Pla+Gem+Bev
Marinis2017 0.275 0.07060011 9.9% 0.28 [0.14, 0.41]
Subtotal (95% CI) 9.9% 0.28 [0.14, 0.41]
Heterogeneity: Not applicable
Test for overall effect: Z = 3.90 (P < 0.0001)
7.1.5 Pla+Pem+Bev
Schuette2017 0.56716418 0.04280195 10.1% 0.57 [0.48, 0.65]
Subtotal (95% CI) 10.1% 0.57 [0.48, 0.65]
Heterogeneity: Not applicable
Test for overall effect: Z = 13.25 (P < 0.00001)
7.1.6 Pem+Bev
Fukuda2019 0.75 0.09682458 9.7% 0.75 [0.56, 0.94]
Schuette2017 0.37815126 0.04445306 10.1% 0.38 [0.29, 0.47]
Subtotal (95% CI) 19.8% 0.55 [0.19, 0.92]
Heterogeneity: Tau² = 0.06; Chi² = 12.18, df = 1 (P = 0.0005); I² = 92%
Test for overall effect: Z = 2.98 (P = 0.003)
7.1.7 Gem+Bev
Marinis2017 0.04651163 0.03211473 10.2% 0.05 [-0.02, 0.11]
Subtotal (95% CI) 10.2% 0.05 [-0.02, 0.11]
Heterogeneity: Not applicable
Test for overall effect: Z = 1.45 (P = 0.15)
Total (95% CI) 100.0% 0.57 [0.32, 0.82]
Heterogeneity: Tau² = 0.16; Chi² = 892.26, df = 9 (P < 0.00001); I² = 99%
Test for overall effect: Z = 4.50 (P < 0.00001)
Test for subgroup differences: Chi² = 267.89, df = 6 (P < 0.00001), I² = 97.8%
-2 -1 0 1 2
C
Study or Subgroup Event Rate SE Weight Event Rate IV, Random, 95% CI Event Rate IV, Random, 95% CI
7.2.1 Pla+Pac+Endo
Han2011 0.30645161 0.05854954 6.6% 0.31 [0.19, 0.42]
Subtotal (95% CI) 6.6% 0.31 [0.19, 0.42]
Heterogeneity: Not applicable
Test for overall effect: Z = 5.23 (P < 0.00001)
7.2.2 Pla+Gem+Endo
Yu2018 0.04166667 0.04078938 6.8% 0.04 [-0.04, 0.12]
Subtotal (95% CI) 6.8% 0.04 [-0.04, 0.12]
Heterogeneity: Not applicable
Test for overall effect: Z = 1.02 (P = 0.31)
7.2.3 Pla+Pem+Endo
Zhou2018 0.14285714 0.04676098 6.7% 0.14 [0.05, 0.23]
Subtotal (95% CI) 6.7% 0.14 [0.05, 0.23]
Heterogeneity: Not applicable
Test for overall effect: Z = 3.06 (P = 0.002)
7.2.4 Pla+Pac+Bev
Boutsikou2013 0.07142857 0.03441515 6.8% 0.07 [0.00, 0.14]
Galetta2014 0.25862069 0.05749603 6.6% 0.26 [0.15, 0.37]
Johnson2004 0.20588235 0.06934458 6.5% 0.21 [0.07, 0.34]
Niho2011 0.71896956 0.02175294 6.9% 0.72 [0.68, 0.76]
Soria2011 0.16666667 0.05750546 6.6% 0.17 [0.05, 0.28]
Subtotal (95% CI) 33.5% 0.29 [-0.04, 0.61]
Heterogeneity: Tau² = 0.14; Chi² = 320.85, df = 4 (P < 0.00001); I² = 99%
Test for overall effect: Z = 1.72 (P = 0.09)
7.2.5 Pem+Bev
Fukuda2019 0.8 0.08944272 6.3% 0.80 [0.62, 0.98]
Schuette2017 0.2184874 0.0378798 6.8% 0.22 [0.14, 0.29]
Spigel2018 0.15254237 0.04680888 6.7% 0.15 [0.06, 0.24]
Subtotal (95% CI) 19.8% 0.38 [0.10, 0.65]
Heterogeneity: Tau² = 0.06; Chi² = 42.84, df = 2 (P < 0.00001); I² = 95%
Test for overall effect: Z = 2.66 (P = 0.008)
7.2.6 Pla+Gem+Bev
Marinis2017 0.375 0.07654655 6.4% 0.38 [0.22, 0.53]
Subtotal (95% CI) 6.4% 0.38 [0.22, 0.53]
Heterogeneity: Not applicable
Test for overall effect: Z = 4.90 (P < 0.00001)
7.2.7 Pla+Pem+Bev
Schuette2017 0.51492537 0.04317417 6.8% 0.51 [0.43, 0.60]
Spigel2018 0.41818182 0.06651121 6.6% 0.42 [0.29, 0.55]
Subtotal (95% CI) 13.3% 0.48 [0.39, 0.57]
Heterogeneity: Tau² = 0.00; Chi² = 1.49, df = 1 (P = 0.22); I² = 33%
Test for overall effect: Z = 10.31 (P < 0.00001)
7.2.8 Gem+Bev
Marinis2017 0.11627907 0.04888483 6.7% 0.12 [0.02, 0.21]
Subtotal (95% CI) 6.7% 0.12 [0.02, 0.21]
Heterogeneity: Not applicable
Test for overall effect: Z = 2.38 (P = 0.02)
Total (95% CI) 100.0% 0.30 [0.15, 0.44]
Heterogeneity: Tau² = 0.08; Chi² = 541.25, df = 14 (P < 0.00001); I² = 97%
Test for overall effect: Z = 4.07 (P < 0.0001)
Test for subgroup differences: Chi² = 65.17, df = 7 (P < 0.00001), I² = 89.3%
-1 -0.5 0 0.5 1
D
Study or Subgroup Event Rate SE Weight Event Rate IV, Random, 95% CI Event Rate IV, Random, 95% CI
7.4.1 Pla+Pac+Endo
Han2011 0.14516129 0.04473749 7.2% 0.15 [0.06, 0.23]
Subtotal (95% CI) 7.2% 0.15 [0.06, 0.23]
Heterogeneity: Not applicable
Test for overall effect: Z = 3.24 (P = 0.001)
7.4.2 Pla+Pem+Endo
Zhou2018 0.85714286 0.04676098 7.1% 0.86 [0.77, 0.95]
Subtotal (95% CI) 7.1% 0.86 [0.77, 0.95]
Heterogeneity: Not applicable
Test for overall effect: Z = 18.33 (P < 0.00001)
7.4.3 Pla+Pac+Bev
Galetta2014 0.01724138 0.0170921 7.6% 0.02 [-0.02, 0.05]
Johnson2004 0.23529412 0.0727467 6.5% 0.24 [0.09, 0.38]
Niho2011 0.36134454 0.04403729 7.2% 0.36 [0.28, 0.45]
Spigel2012 0.1 0.04743416 7.1% 0.10 [0.01, 0.19]
Subtotal (95% CI) 28.4% 0.18 [0.00, 0.35]
Heterogeneity: Tau² = 0.03; Chi² = 58.82, df = 3 (P < 0.00001); I² = 95%
Test for overall effect: Z = 1.97 (P = 0.05)
7.4.4 Pem+Bev
Fukuda2019 0.15 0.0798436 6.3% 0.15 [-0.01, 0.31]
Schuette2017 0.18487395 0.0355858 7.3% 0.18 [0.12, 0.25]
Spigel2018 0.15254237 0.04680888 7.1% 0.15 [0.06, 0.24]
Subtotal (95% CI) 20.8% 0.17 [0.12, 0.22]
Heterogeneity: Tau² = 0.00; Chi² = 0.38, df = 2 (P = 0.83); I² = 0%
Test for overall effect: Z = 6.38 (P < 0.00001)
7.4.5 Pla+Gem+Bev
Marinis2017 0.1 0.04743416 7.1% 0.10 [0.01, 0.19]
Reck2009 0.09422492 0.01610628 7.6% 0.09 [0.06, 0.13]
Subtotal (95% CI) 14.7% 0.09 [0.06, 0.12]
Heterogeneity: Tau² = 0.00; Chi² = 0.01, df = 1 (P = 0.91); I² = 0%
Test for overall effect: Z = 6.22 (P < 0.00001)
7.4.6 Pla+Pem+Bev
Schuette2017 0.26119403 0.03794848 7.3% 0.26 [0.19, 0.34]
Spigel2018 0.14545454 0.04753898 7.1% 0.15 [0.05, 0.24]
Subtotal (95% CI) 14.4% 0.21 [0.09, 0.32]
Heterogeneity: Tau² = 0.00; Chi² = 3.62, df = 1 (P = 0.06); I² = 72%
Test for overall effect: Z = 3.58 (P = 0.0003)
7.4.7 Gem+Bev
Marinis2017 0.06976744 0.03884972 7.3% 0.07 [-0.01, 0.15]
Subtotal (95% CI) 7.3% 0.07 [-0.01, 0.15]
Heterogeneity: Not applicable
Test for overall effect: Z = 1.80 (P = 0.07)
Total (95% CI) 100.0% 0.20 [0.11, 0.30]
Heterogeneity: Tau² = 0.03; Chi² = 337.86, df = 13 (P < 0.00001); I² = 96%
Test for overall effect: Z = 4.24 (P < 0.0001)
Test for subgroup differences: Chi² = 246.88, df = 6 (P < 0.00001), I² = 97.6%
-1 -0.5 0 0.5 1

Figure S3

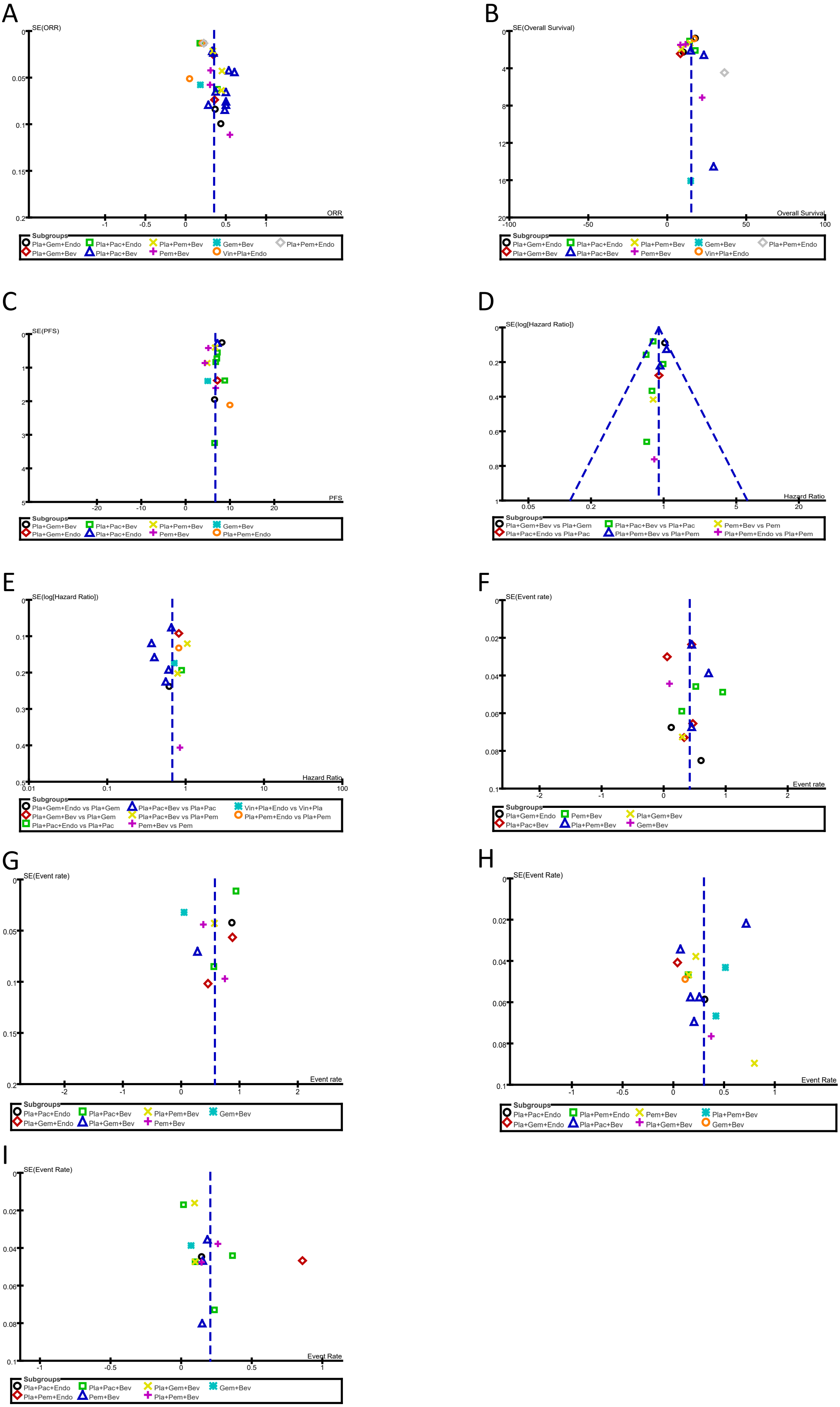
A
SE(ORR)
ORR
Subgroups
Pla+Gem+Endo
Pla+Gem+Bev
Pla+Pac+Endo
Pla+Pac+Bev
Pla+Pem+Bev
Pem+Bev
Gem+Bev
Vin+Pla+Endo
Pla+Pem+Endo
B
SE(Overall Survival)
Overall Survival
C
SE(PFS)
PFS
D
SE(log[Hazard Ratio])
Hazard Ratio
Pla+Gem+Bev vs Pla+Gem
Pla+Pac+Endo vs Pla+Pac
Pla+Pac+Bev vs Pla+Pac
Pla+Pem+Bev vs Pla+Pem
Pem+Bev vs Pem
Pla+Pem+Endo vs Pla+Pem
E
SE(log[Hazard Ratio])
Hazard Ratio
Pla+Gem+Endo vs Pla+Gem
Pla+Gem+Bev vs Pla+Gem
Pla+Pac+Endo vs Pla+Pac
Pla+Pac+Bev vs Pla+Pac
Pla+Pac+Bev vs Pla+Pem
Pem+Bev vs Pem
Vin+Pla+Endo vs Vin+Pla
Pla+Pem+Endo vs Pla+Pem
F
SE(Event rate)
Event rate
G
SE(Event rate)
Event rate
H
SE(Event Rate)
Event Rate
I
SE(Event Rate)
Event Rate
